# USP33 Regulates DNA Damage Response and Carcinogenesis Through Deubiquitylating and Stabilising p53

**DOI:** 10.1111/cpr.13793

**Published:** 2024-12-18

**Authors:** Yuqi Zhu, Zixiang Chen, Kaifeng Niu, Mengge Li, Yuchun Deng, Ji Zhang, Di Wei, Jiaqi Wang, YongLiang Zhao

**Affiliations:** ^1^ China National Center for Bioinformation Beijing China; ^2^ Beijing Institute of Genomics Chinese Academy of Sciences Beijing China; ^3^ University of Chinese Academy of Sciences Beijing China

## Abstract

The de‐ubiquitinase USP33 has been shown to possess either tumour‐promoting or inhibitory effect on human cancer cells. However, all these findings are mainly based on in vitro cell culture models, and the in vivo evidence, which is more plausible to digest the functional role of USP33 in carcinogenic process, is still lacking. Here, we demonstrate that USP33 modulates DNA damage responses including cell cycle arrest and apoptosis induction through associating with p53. It directly interacts with p53 to mediate its de‐ubiquitination and further  stabilisation under DNA damage condition. Depletion of USP33 induces an enhanced level of p53 ubiquitination, which de‐stabilises p53 protein leading to impaired DNA damage responses. Furthermore, USP33 silencing shows either promoted or inhibited effect on cell proliferation in human cancer cells with p53 WT and mutant background, respectively. Consistently, mice with hepatocyte‐specific USP33 knockout are more sensitive to nitrosodiethylamine (DEN)‐induced hepatocarcinogenesis compared to wild type mice. Thus, our in vitro and in vivo evidences illustrate that USP33 possesses anti‐tumour activity via regulating p53 stability and activity.

## Graphical abstract

Our findings demonstrated that USP33 deubiquitinates p53 and regulates its stability, and moreover, DNA damage can stabilise p53 through enhancing its association with USP33, leading to strong DNA damage response (DDR). However, USP33 deficiency results in defective DDR, facilitating DEN‐induced hepatocarcinogenesis in hepatocyte‐specific USP33 KO mice.

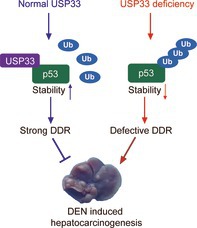

## 1 Introduction

USP33, also known as the VHL‐interacting deubiquitinating enzyme 1 (VDU1), has been shown to plays important roles in a variety of cellular activities, including centrosomal replication, thyroid hormone activation, immunity, mitophagy and cell migration by modulating the stability of multiple proteins through deubiquitination [1, 2, 3]. However, current reports illustrate a controversial role of USP33 in human cancer cells. For instance, USP33 was shown to inhibit cell migration through deubiquitinating and stabilising roundabout homologue 1 (ROBO1), thus functioning as a tumour suppressor in lung cancer cells [4]. In contrary, an oncogenic role of USP33 was also observed in hepatocellular carcinoma (HCC) and prostate cancer cells [5, 6]. USP33 directly bound dual specificity protein phosphatase 1 (DUSP1) and reduced its ubiquitination, thereby impairing JNK activation and apoptosis in prostate cancer [7]. Moreover, USP33 could inhibit the ubiquitination of transcription factor Sp1 (SP1) which led to upregulated c‐Met expression and promoted migration and invasion of HCC [8]. However, all these findings are mainly based on in vitro cancer cell culture models. Therefore, in vivo evidence is definitely required to clarify the function of USP33 in tumorigenic process.

The tumour suppressor P53 is the most commonly mutated gene found in human cancers [9] and possesses the capability to respond to genotoxic stress signals, which protects cells and organisms from genomic instability caused by DNA damage and is considered the primary mechanism by which p53 prevents tumour development [10]. A large number of posttranslational modifications on p53 have been shown to modulate its stability and activity, including phosphorylation, acetylation, methylation, glycosylation, and ubiquitination, amongst which ubiquitination plays an important role [11, 12]. In unstressed cells, p53 is maintained at low levels due to the activity of the RING‐finger E3 ubiquitin ligase MDM2 [13], or other MDM2‐independent pathways mediated by different E3 ligases such as PIRH2, COP1, ARF‐BP1, and CHIP [14, 15]. As the counteracting enzyme of E3 ligases, several deubiquitinating enzymes (DUBs) have been reported to directly or indirectly regulate the ubiquitination of p53, especially under cellular stresses [16]. For example, USP10 was phosphorylated by ATM under DNA damage, and was then recruited to p53 leading to its de‐ubiquitination and stabilisation [17]. Similarly, OTUB1 was shown to be capable of deubiquitinating p53 and mediating its stabilisation [18].

To further clarify the potential function of USP33 in tumorigenic process, we employed in vitro cell culture and USP33 conditional knockout mouse models to determine the potential significance of USP33 in tumour progression. We found that USP33 serves as the DUB of p53, and through stabilising its protein level displays protective roles under DNA damage stress. Interestingly, depending on the mutational status of p53, USP33 depletion presents differential roles in regulating HCC cell proliferation and tumorigenicity. In particular, mice with conditional USP33 KO in hepatocyte are significantly prone to hepatocarcinogenesis induced by nitrosodiethylamine (DEN) in relative to wild type (WT) and heterozygous mice. Thus, our results clearly reveal that USP33 exerts anti‐tumour effect under DNA damage stress through maintaining p53 stability.

## 2 Results

### 2.1 USP33 Interacts With Tumour Suppressor p53

**FIGURE 1:**
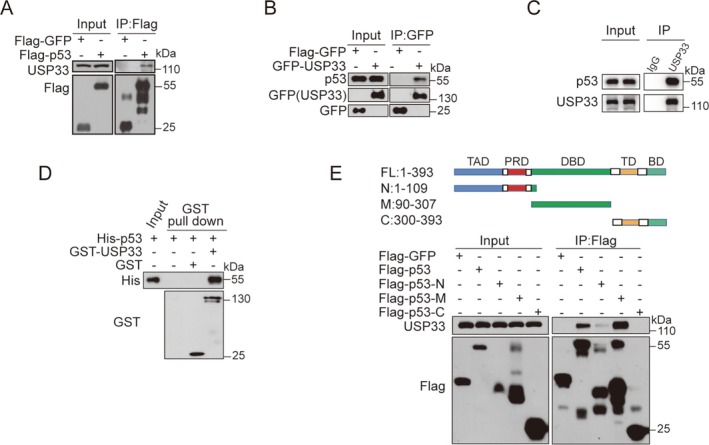
USP33 interacts with p53. (A) Flag‐p53 interacts with endogenous USP33. (B) GFP‐USP33 interacts with endogenous p53. (C) Endogenous association between USP33 and p53. (D) USP33 interacts with p53 in vitro. (E) USP33 interacts with the N and M domain of p53.

In an effort to search for the downstream effector of USP33, we found p53 protein level was substantially decreased upon USP33 knockdown, which drives us to explore the association of USP33 and p53 under pathophysiological conditions. First, their interaction was validated by transfecting Flag‐p53 into 293T cells, followed by co‐immunoprecipitation (co‐IP) assays. Endogenous USP33 was found to be co‐precipitated by the Flag‐p53 (Figure 1A). Likewise, endogenous p53 can be co‐precipitated by the GFP‐USP33 (Figure 1B). To further verify the interaction between p53 and USP33, 293T whole cell lysates were incubated with control IgG or anti‐USP33 antibody, followed by Western blotting analysis on the pull‐down complex. Endogenous p53 was found to be co‐precipitated with USP33, but not with control IgG (Figure 1C). We then performed GST‐pulldown assay, and the results confirmed a direct interaction between p53 and USP33 (Figure 1D).

We further determined which domains of p53 interacts with USP33 by constructing different functional domains of Flag‐p53‐N, Flag‐p53‐M and Flag‐p53‐C, and found that both Flag‐p53‐N, Flag‐p53‐M, instead of Flag‐p53‐C, interact with USP33 (Figure 1E).

### 2.2 USP33 Deubiquitinates p53 and Regulates Its Stability

**FIGURE 2:**
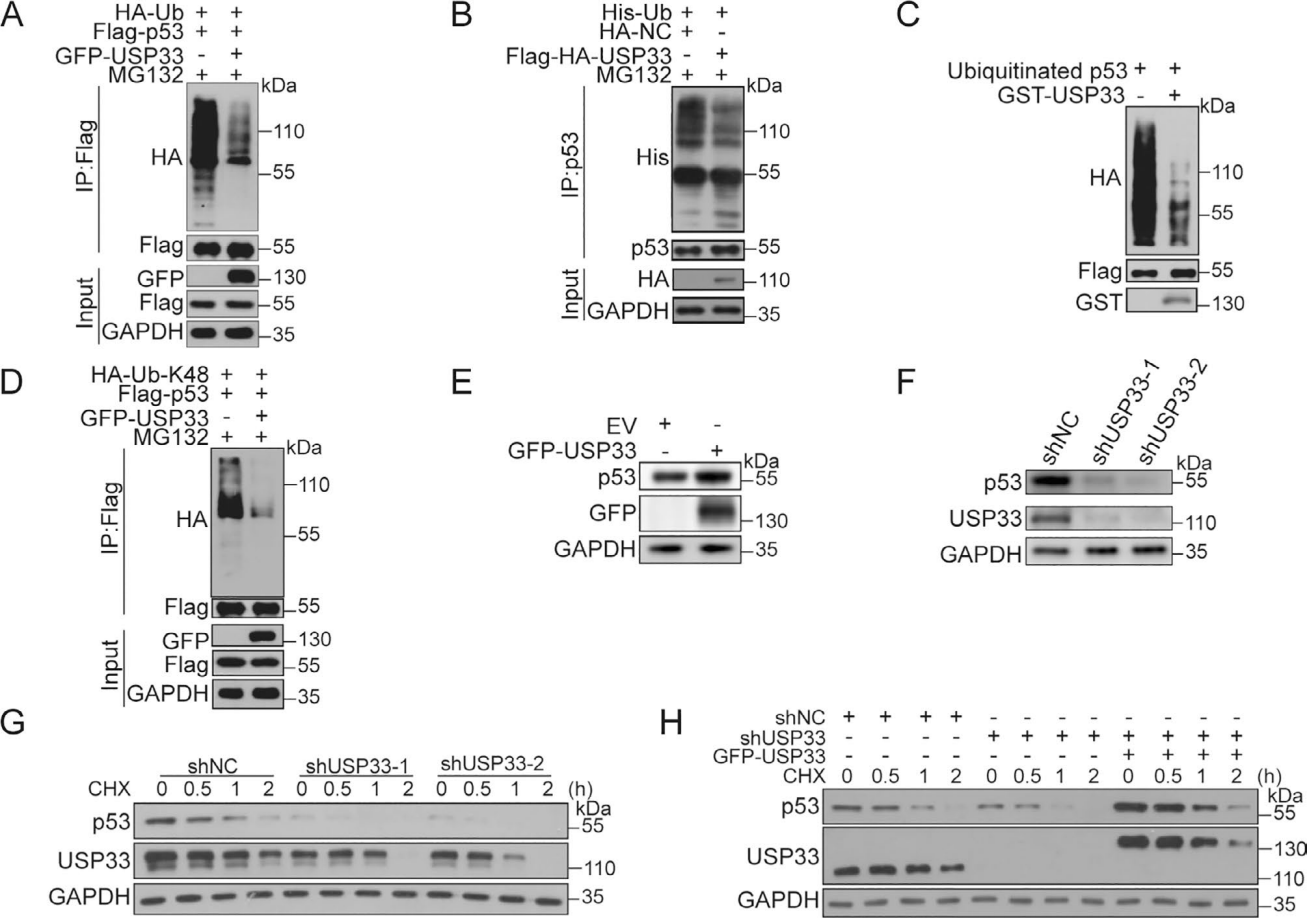
USP33 deubiquitinates p53 and regulates its stability. (A) USP33 deubiquitinates Flag‐p53 in cells. (B) USP33 deubiquitinates p53 in cells. (C) USP33 deubiquitinates p53 in vitro. (D) USP33 reduced K48 ubiquitination level of p53. (E) Overexpression of USP33 enhances p53 protein level in cells. (F) Knocking down of USP33 decreases p53 protein level in cells. (G) Knocking down of USP33 decreases p53 stability. (H) Knocking down of USP33 decreases p53 stability can be rescued by overexpression of USP33. Data were showed as mean ± standard error of mean (SEM) from at least three independent experiments. Statistical analysis by one‐way or two‐way ANOVA. *p ≤ 0.05, **p ≤ 0.01.

The findings of USP33 directly interacting with p53 drove us to determine whether USP33 deubiquitinates p53 and further regulates its stability. To this end, 293T cells were co‐transfected with constructs of Flag‐p53, GFP‐USP33 and HA‐Ub, and further treated with the proteasome inhibitor MG132 before being harvested. The results showed that the ubiquitination of Flag‐p53 was markedly reduced by co‐transfection of GFP‐USP33 (Figure 2A). Similarly, endogenous p53 ubiquitination levels was also significantly diminished by overexpression of GFP‐USP33 (Figure 2B). An in vitro ubiquitination assay was next carried out to explore the direct deubiquitination activity of USP33 towards p53. GST‐USP33 purified from bacteria and ubiquitinated p53 protein from 293T cells were used. As shown in Figure 2C, GST‐USP33 effectively deubiquitinated p53 in vitro.

As ubiquitin K48 is the main form of branched chain mediating protein degradation [19], we next determined whether USP33 could diminish the ubiquitin K48 branch chain of p53. Flag‐p53, GFP‐USP33 and HA‐Ub‐K48 were co‐transfected into 293T cells followed by co‐IP and Western blotting analyses. A significantly reduced K48 ubiquitination level of p53 was observed when USP33 was overexpression (Figure 2D), indicating that USP33 stabilises p53 mainly through deubiquitinating the K48 branch chain of p53.

We further examine the effect of USP33‐mediated deubibiquitination on p53 protein stability. As shown in Figure 2A, USP33 overexpression in U2OS cells markedly increased the endogenous p53 levels (Figure 2E). On the contrast, knockdown of USP33 by shRNA decreased the levels of endogenous p53 in U2OS cells (Figure 2F). A CHX assay was further performed to confirm the effect of USP33 on p53 stability. Both control or USP33 knockdown U2OS cells were treated with CHX for different periods of time and verified by Western blotting. The result showed that p53 stability was substantially decreased upon USP33 knockdown (Figure 2G). More importantly, the decreased p53 stability was mostly reversed when USP33 was re‐expressed in USP33 knockdown U2OS cells (Figure 2H). These results illustrate that USP33 can regulate the stability of p53 protein through mediating its de‐ubiquitination.

### 2.3 USP33 Regulates p53‐Dependent DNA Damage Response

**FIGURE 3:**
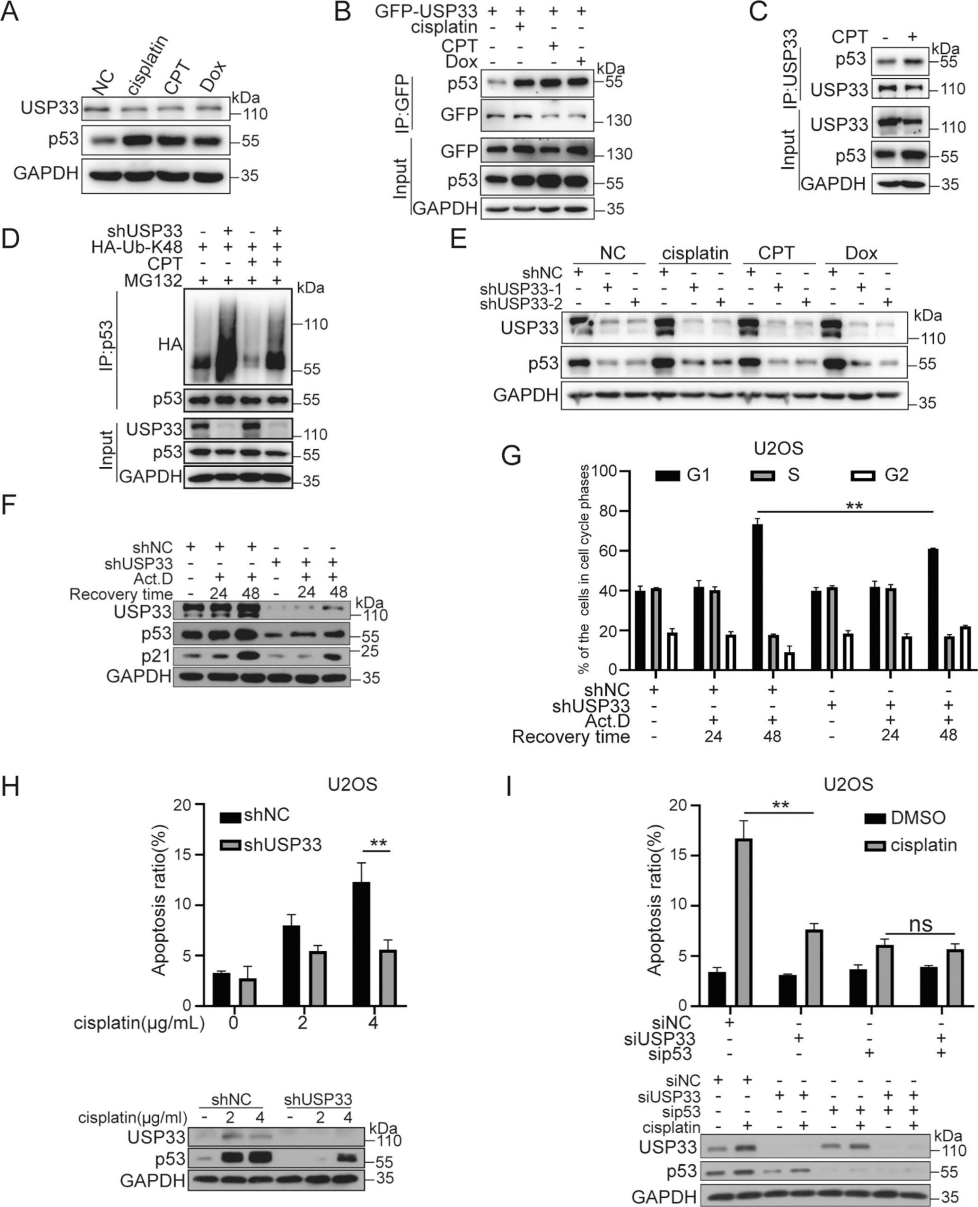
USP33 regulates p53‐dependent DNA damage response. (A) DNA damage enhances p53 protein level in cells. (B) DNA damage increases the interaction between GFP‐USP33 and p53. (C) The interaction between endogenous USP33 and p53 was enhanced by CPT. (D) Knocking down of USP33 abolished the decreased of K48 ubiquitination of p53 induced by CPT. (E) Knocking down of USP33 inhibits the increases of p53 induced by DNA damage. (F) Knocking down of USP33 inhibits the increases of p21 induced by DNA damage. (G) Knocking down of USP33 inhibits cell cycle arrest. (H) Knocking down of USP33 inhibits apoptosis. (I) USP33 regulates apoptosis depend on p53. Data were showed as mean ± standard error of mean (SEM) from at least three independent experiments. Statistical analysis by one‐way or two‐way ANOVA. *p ≤ 0.05, **p ≤ 0.01.

As a genome guardian, p53 is stabilised after DNA damage to maintain genome stability and determine cell fate [20]. We then determine whether USP33 regulates p53 stability and activation after DNA damage. As expected, the expression of p53 was upregulated after treatment with DNA damage agents including cisplatin, CPT and Dox in U2OS cells, while USP33 expression was unchanged (Figure 3A). We then examined the association of USP33 with p53 under DNA damage condition. GFP‐USP33 was overexpressed in U2OS cells, followed by the treatment with DNA damage agents such as cisplatin, CPT and Dox. The result showed that the interaction between USP33 and p53 was substantially enhanced upon cisplatin, CPT or Dox treatment (Figure 3B). To further verify this result, an endogenous interaction was tested by pulling down the endogenous USP33 using anti‐USP33 antibody. Consistently, the interaction between USP33 and p53 showed a marked enhancement after CPT treatment (Figure 3C). In support, USP33 knockdown markedly enhanced the K48 ubiquitination level of p53, while CPT treatment markedly decreased the K48 ubiquitination level. Moreover, this effect was more pronounced in shCon cells than in USP33 knockdown cells (Figure 3D). Meanwhile, depletion of USP33 by shRNA markedly reduced p53 proteins levels under the above treatments in U2OS cells (Figure 3E). These results strongly support that USP33 regulates p53 stability in response to DNA damage through an enhanced association.

p53 is an important tumour suppressor involving the regulation of DNA damage responses including cell proliferation, apoptosis and cell cycle arrest through a series of downstream molecules [11]. As USP33 promotes p53 stability under DNA damage condition, we then examined the potential significance of USP33 in DNA damage response. U2OS cells were depleted with USP33 expression and then treated with 5 nM Act D for 2 h, followed by recovery with indicated time‐points. In relative to shControl cells, USP33 silencing markedly decreased the protein levels of both p53 and p21 (Figure 3F). Meanwhile, G1‐phase cell cycle arrest in shControl cells were also mostly abolished post USP33 depletion (Figure 3G). To further determine whether USP33 regulates apoptotic response in a p53‐dependent manner, cisplatin‐induced apoptotic response in U2OS and BEL7402 human hepatocarcinoma cells with or without USP33 depletion was examined by Flow cytometry. As shown in Figures 3H and S1, USP33 knockdown significantly decreased the percentage of apoptotic cells, while this effect was lost when p53 expression was concurrently silenced with USP33 (Figure 3I). These data suggest that p53 acts as the downstream effector required for USP33‐regulated cycle arrest and apoptotic response under DNA damage condition.

### 2.4 USP33 Differentially Regulates Cell Proliferation and Tumorigenic Potential Dependent of p53 Status

**FIGURE 4:**
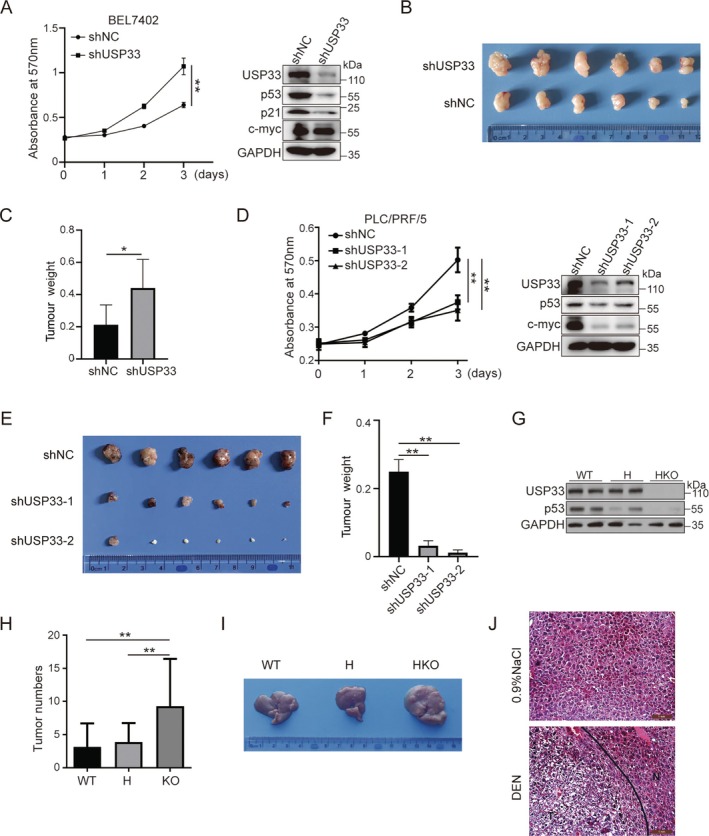
USP33 regulates cell proliferation and DEN‐induced hepatocarcinogenesis. (A) Knocking down of USP33 by shUSP33‐1 + shUSP33‐2 enhances cell proliferation in BEL7402 cells. (B) Comparison of subcutaneous tumours induced in nude mice by subcutaneous injection with BEL7402 cells from two groups. (C) Tumour weight in nude mice injected with BEL7402 cells from two groups. (D) Knocking down of USP33 inhibits cell proliferation in PLC/PRF/5 cells. (E) Comparison of subcutaneous tumours induced in nude mice by subcutaneous injection with PLC/PRF/5 cells from three groups. (F) Tumour weight in nude mice injected with PLC/PRF/5 cells from three groups. (G) hepatocyte‐specific USP33 knockout decreases p53 level in liver tissues. (H) Number of visible liver tumours of liver tumours. (I) Representative images of liver tumours in the USP33 WT, Heterozygous (H) and HKO mice after DEN injection. (J) Representative images of H&E stained liver. Data were showed as mean ± standard error of mean (SEM) from at least three independent experiments. Statistical analysis by *t*‐test, one‐way or two‐way ANOVA. *p ≤ 0.05, **p ≤ 0.01.

p53 serves as a crucial tumour suppressor involved in controlling cell proliferation and tumour initiation [21]. As USP33 enhances p53 stability through deubiquitination, it is plausible that USP33 could also elicit anti‐tumour activity. In support, USP33 knockdown by shRNA in BEL7402 hepatocarcinoma cells (p53‐WT) significantly promoted not only cell proliferation (Figure 4A) but also the growth of xenograft tumours in nude mice (Figure 4B,C).

USP33 can also stabilise the mutant p53 protein (Figure S2), and p53 gain‐of‐function mutant (p53‐R249S) has been shown to drive PLC/PRF/5 HCC cell proliferation and tumorigenesis by stabilising c‐myc [22, 23]. We then used this cell line to determine the effect of USP33 in tumorigenicity. In contrast to the effect of USP33 knockdown in BEL7402 cells, USP33 silencing in PLC/PRF/5 cells significantly inhibited cell proliferation with decreased protein levels of both p53 and c‐myc (Figure 4D), and meanwhile, tumorigenic ability in nude mice was also significantly decreased in USP33 knockdown PLC/PRF/5 cells in relative to PLC/PRF/5 shRNA control cells (Figure 4E,F). These data clearly indicate that USP33 differentially regulates HCC cell proliferation and tumorigenicity dependent on p53 and its mutational status.

### 2.5 Mice With USP33 Knockout are Sensitive to DEN‐Induced Hepatocarcinogenesis

Due to USP33 inhibit cell proliferation and tumorigenicity in liver cancer cells with WT p53, we employed a conditional USP33 knockout mouse model to determine whether USP33 null renders mice more prone to tumorigenesis. We generated a hepatocyte‐specific USP33 knockout mouse (USP33‐HKO) in which the coding region, exon 6 of the USP33 gene was deleted using a Cre‐loxP system (Figure S3A,B). The authenticity of USP33 knockout was confirmed by genomic PCR and Western blot analyses (Figure S3C,D). No obvious differences in the development, health and life span were observed in the knockout mice in comparison with their WT littermates. We then treated the littermates of USP33 WT, heterozygous and HKO mice with nitrosodiethylamine (DEN) to specifically induce HCC development [24]. In relative to WT and heterozygous mice, live tissue in USP33‐null mice showed a markedly lower p53 expression level under DEN treatment (Figure 4G). At end of 8‐month experimental period, the mice were sacrificed and tumour nodules on the liver surface were counted. The results showed that the number of nodules on the liver surface of USP33 HKO mice was significantly higher than that of wild‐type and heterozygous mice (Figure 4H,I). The liver tissues of DEN‐induced and control mice were sliced and stained by HE. The results showed that the liver of DEN‐induced mice had obvious pathological changes (Figure 4J). The above experiments confirmed that conditional USP33 deficiency in liver tissue promotes DEN‐induced hepatocarcinogenesis in mice.

## 3 Discussion

The current reported findings in different tissue types of human cancer cell lines show a controversial effect of USP33 on tumorigenicity, suggesting the existence of unidentified molecular mechanism(s) that mediate USP33‐regulated tumorigenic phenotype. In this study, we provide strong evidence that USP33 directly targets and stabilises p53 tumour suppressor under DNA damage stress condition, and presents anti‐tumour effect supported by the findings that USP33 regulates p53‐dependent DNA damage responses including cell‐cycle arrest and apoptotic induction, and loss of its expression not only promotes HCC cell proliferation and tumorigenicity in nude mice but also accelerates hepatocarcinogenesis induced by DEN in conditional hepatocytic USP33 KO mice. It is worth noting that USP33 can also target mutated p53, especially p53‐R249S that has been shown to have a gain‐of‐function in promoting HCC cell proliferation and tumorigenicity [23]. USP33 depletion can de‐stabilise p53‐R249S, resulting in the suppression of HCC cell proliferation and tumorigenic potential. Our in vitro and in vivo findings convincingly demonstrate the anti‐tumour effect of USP33. However, the trait of genetic background/downstream targets, such as p53‐R249S, may perplex its regulatory modes likely with totally contradictory effects. Nevertheless, in human cancers carrying p53‐R249S, USP33 might represent a valuable target to improve the clinical outcome of cancer patients.

p53 employs multiple pathways to maintain genomic stability [25]. On the one hand, p53 upregulates the cell cycle inhibitor p21 (Cdkn1a), leading to cell cycle arrest and enabling DNA repair to occur [26, 27]. On the other hand, p53 activates pro‐apoptotic genes such as Bax, Noxa and Puma to eliminate cells with unrepairable DNA damages [20]. In addition, p53 serves as a transcription factor to transcriptionally regulate hundreds of genes involved in a variety of cellular processes, including metabolism, redox biology, stemness and cell fate eta [5, 28, 29, 30]. Several mechanisms have been reported to regulate the enhanced stability of p53 after DNA damage [6]. For example, p53 was phosphorylated by ATM and Chk2, which led to a decreased interaction between p53 and MDM2 [31, 32]. Furthermore, following DNA damage, USP10 translocates to the nucleus and deubiquitinate p53, thereby stabilising and activating p53. Our study further identified USP33 as the critical regulator for p53 stabilisation. In particular, DNA damage agents can enhance the association of USP33 and p53 resulting in the removal of K48 polyubiquitination on p53 and consequent protein stabilisation. Loss of USP33 induces p53 de‐stabilisation and further defective p53‐dependent DNA damage responses. Thus, USP33 might cooperate with other deubiquitinases to stabilise p53 protein under DNA damage stress condition. But their detailed regulatory modes are worthy of further investigations.

The major risk factors for HCC are complex and multifaceted, including hepatitis virus infection, alcohol, nonalcoholic fatty liver disease, chronic metabolic abnormalities, diet contamination and others [33]. All chronic liver diseases caused by various etiologies are associated with genomic instability, and these changes can be detected even before atypical hyperplasia occurs [34]. Moreover, patients with liver cancer often exhibit increased sensitivity to DNA damage [35], indicating that genomic instability plays a role in the development and progression of liver cancer. Additionally, HCC is classified as a “genomically unstable” cancer due to typical manifestations such as chromosomal breaks, aneuploidy and oxidative DNA damage [36]. In this paper, we established the transgenic mouse model with specific USP33 KO in hepatocyte, which is subjected to DEN treatment for HCC induction. The model of DEN‐induced mouse liver cancer has been well‐established [23], where DEN was bioactivated by cytochrome p450 (CYP) enzymes present in liver to form alkalating adducts inducing hepatocellular carcinoma (HCC). Based on the data from the USP33 HKO mice, it is evidenced that USP33 KO in hepatocyte decreases p53 protein level which consequently induces genomic instability due to defective DNA damage response and inability to eliminate the cells with DNA damages. The eventual outcome is to make the mice significantly prone to hepatocarcinogenesis induced by DEN.

**FIGURE 5:**
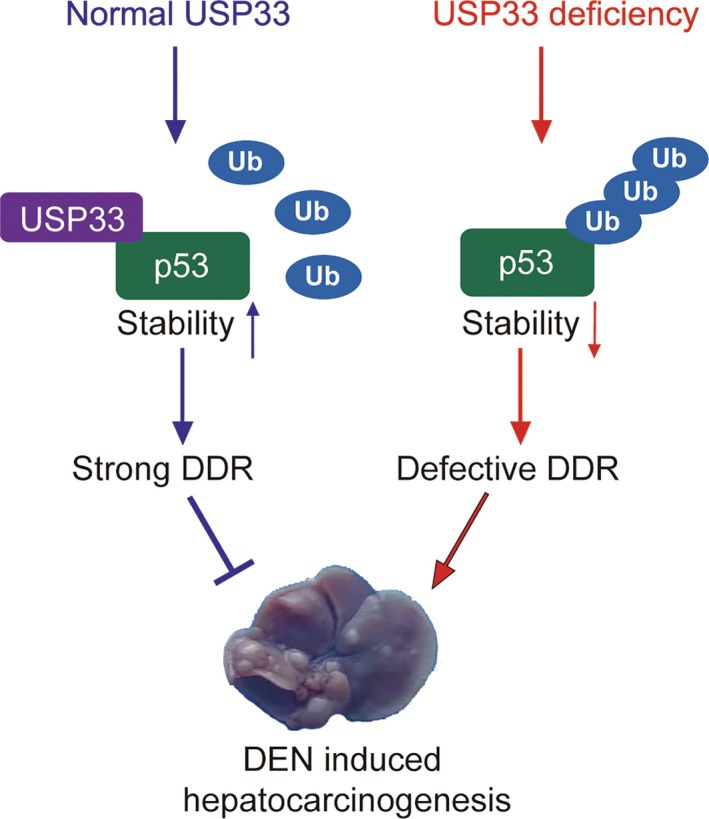
A mechanism of regulation of USP33‐coupled p53 ubiquitination and activation in DNA damage response. USP33 deubiquitinates p53 and regulates its stability, and moreover, DNA damage can stabilise p53 through enhancing its association with USP33, leading to a strong DNA damage response (DDR). However, USP33 deficiency results in defective DDR, facilitating DEN‐induced hepatocarcinogenesis in hepatocyte‐specific USP33 KO mice.

In summary, our findings demonstrated that USP33 deubiquitinates p53 and regulates its stability, and moreover, DNA damage can stabilise p53 through enhancing its association with USP33, leading to a strong DNA damage response (DDR). However, USP33 deficiency results in defective DDR, facilitating DEN‐induced hepatocarcinogenesis in hepatocyte‐specific USP33 KO mice (Figure 5). As p53 is one of the most frequently mutated tumour suppressors in human cancer [15], and meanwhile, the stability of mutated p53 (R249S) protein is equally regulated by USP33, therefore, specific targeting USP33 in a subset of human cancer with p53 mutation (R249S) might yield potential translational value in clinal cancer treatment.

## 4 Materials and Methods

### 4.1 Cell Lines

The U2OS, 293T cells were obtained from the American Type Culture Collection. PLC/PRF/5 and BEL7402 were obtained from Cell Resource Center, Chinese Academy of Medical Sciences. Cells were cultured in DMEM supplemented with 10% (v/v) FBS (B118‐500, Nobimpex), 100 U/mL penicillin and 100 μg/mL streptomycin (15,140,122, Gibco) in a humidified incubator at 37°C with 5% CO_2_. All cell lines were tested as negative for mycoplasma contamination.

### 4.2 Chemicals and Antibodies

The following chemical reagents are used in this paper: MG‐132 (C2211), MTT (ST316), CHX (S7418), cisplatin (15663‐27‐1), Actinomycin D (S8964), CPT (64439‐81‐2), Dox (25316‐40‐9). The antibodies that were used are as follows: anti‐USP33 (NBP1‐82931, Novus Biologicals), anti‐USP33 (20445‐1‐AP, Proteintech), anti‐p53 (DO‐1, Santa Cruz), anti‐p21 (10355‐1‐AP, Proteintech), anti‐GAPDH (MB374, Millipore), anti‐GFP (AE012, Abclonal), anti‐Flag (F3165, sigma), anti‐HA (3724S, Cell Signaling Technology).

### 4.3 Lentiviral‐Mediated shRNA Interference

All shRNAs for USP33 were from the reported literature and purchased from Sangon Biotech.

USP33 shRNA‐1: 5′‐GATCATGTGGCGAAGCATATG‐3′;

USP33 shRNA‐2: 5′‐GGCTTGGATCTTCAGCCATTT‐3′;

Lentivirus particles were produced in HEK293 packaging cells. Then the lentivirus was used for infecting the target cells. Stable cell lines with low expression of USP33 were screened using purinomycin.

### 4.4 Co‐Immunoprecipitation (Co‐IP) Assay

Cells were transfected with indicted expression vector for 48 h, the cells were split using IP buffer (50 mM Tris HCl pH 7.5, 5 mM EDTA, 10 mM EGTA, 0.5% NP‐40, 10% glycerine) for 30 min at 4°C. Then the Flag‐M2 beads or GFP‐agarose were used to enrich the target proteins overnight at 4°C. After washing with IP buffer for three times, the binding complex proteins were eluted from the beads heated at 95°C for 10 min in 1 × Laemmli sample buffer. Western blotting analysis was performed using indicated antibodies.

### 4.5 Protein Purification

The encoding sequences of USP33 and p53 were cloned into the PGX‐6P‐1 and pET‐28b vectors, respectively. Then GST‐USP33 and His‐p53 were expressed in E. coil BL21 (DE3) cells. The protein was induced by 0.5–1 mM IPTG overnight at 16°C. After cracking the bacteria, beads with corresponding tags were used to enrich and purify the proteins. The purified proteins were dissolved in lysis buffer and 20 μL of 50% GST gel suspension for each sample were used. After 4 h rotation at 4°C, the GST gel was washed three times using IP buffer and the sample was heated with 1 × SDS buffer at 95°C for 10 min. The sample was analysed by Western blotting.

### 4.6 In Vivo and In Vitro Ubiquitination Assays

HEK293 cells were transfected with Flag‐p53 and HA‐Ub for 24 h, then transfected with GFP‐USP33. After 48 h, the cells were treated with 20 μM MG132 for 4 h, lysed and immunoprecipitated with Flag M2 beads. The samples were analysed by Western blotting.

For in vitro deubiquitination experiments, purified Flag‐p53 with or without GST‐USP33 were mixture in a deubiquitination buffer (50 mM Tris–HCl pH 8.0, 50 mM NaCl, 1 mM EDTA, 10 mM DTT, 5% glycerol) at 37°C for 2 h. Then the reaction was stopped by heating it with 1 × SDS buffer at 95°C for 10 min. the samples were analysed by Western blotting with the indicated antibodies.

### 4.7 MTT Assay

For the detection of cell viability, 1000 stable cells with or without USP33 knockdown were seeded into each well of 96‐well plates and incubated at 37°C in a CO_2_ incubator (5% CO_2_) for 1, 2, 3 or 4 days. Then treated with 3‐(4, 5‐dimethylthiazol‐2‐yl)‐2,5‐diphenyl tetrazolium bromide (MTT) for 4 h, after DMSO was added to each well and mixing, optical density (OD) at 570 nm was measured using a spectrophotometer.

### 4.8 Cell Cycle Analysis

After harvested, cells were fixed with 75% ethanol for > 2 h at 4°C. Then resuspended in 500 μL of reaction buffer containing 50 μg/mL PI and 100 μg/mL RNase. After mixing, cells were incubated for 30 min in the dark. Cell cycle analysis was measured by flow cytometry.

### 4.9 Apoptosis Assay

After harvested, cells were fixed with Annexin‐V and 7‐AAD following the manufacturer's instructions. Cell apoptosis analysis was measured by flow cytometry.

### 4.10 In Vivo Tumorigenicity Assay

Male C57BL nude mice (5 weeks old) were purchased from Vital River Laboratory Animal Technology (Beijing, China). 5 × 10^6^ stable BEL7402 cells with or without USP33 knockdown or 10^7^ stable PLC/PRF/5 cells with or without USP33 knockdown were injected subcutaneously into nude mice. Mice were sacrificed with euthanasia techniques when the tumours reach the appropriate size. This experiment was approved by the Animal Care and Ethics Committee of China National Center for Bioinformation (Beijing Institute of Genomics, Chinese Academy of Sciences) with approval no. of 2021A008.

### 4.11 DEN‐Induced Liver Tumorigenesis

USP33 flox mice and Alb‐Cre mice (003574 from Jackson Laboratory) were purchased from cyagen. Neonatal of male 14‐day‐old USP33 WT, heterozygous and HKO littermates were subjected to a single intraperitoneal administration of 25 μg/g body weight of the diethylnitrosamine dissolved in saline. Mice were sacrificed with euthanasia techniques at 8 months after birth. Immediately after euthanasia, the livers were excised and photographed. Grossly visible surface liver tumours were scored from the top and bottom of each liver. This experiment was approved by the Animal Care and Ethics Committee of China National Center for Bioinformation (Beijing Institute of Genomics, Chinese Academy of Sciences) with approval no. of 2017A009.

### 4.12 Statistical Analyses

GraphPad Prism 8 was used for all statistical analyses. The data were expressed as mean ± standard deviation (SD), from at least three independent experiments. A Student's *t*‐test was used to compare two groups affected by one single variable. One‐way ANOVA or two‐way ANOVA with Tukey's test was used to compare multiple data groups affected by one or two independent variables, respectively. *p* < 0.05 was considered statistically significant.

## Author Contributions

Y.L.Z., Y.Q.Z., Z.X.C. conceived the project. Y.Q.Z, Z.X.C., K.F.N., M.G.L.,Y.C.D., J.Z., D.W. and J.QW. performed the experiments and analysed the data. Z.C. wrote the manuscript. Y.Z. revised the manuscript.

## Conflicts of Interest

The authors declare no conflicts of interest.

## Supporting information


**Figure S1.** Knocking down of USP33 inhibits apoptosis in BEL7402.
**Figure S2.** Knocking down of USP33 decreases the stability of mutant p53 R249S.
**Figure S3.** Generation of hepatocyte‐specific USP33 knockout mouse (USP33‐HKO) strains. (A) Schematic diagram of generating USP33 knockout mice. (B) Breeding strategies for producing USP33 HKO mice. (C) Genotyping of wild‐type, heterozygous KO and HKO mice using PCR. (D) Western blot was performed to analyse USP33 knockout mice in liver tissues.

## Data Availability

All datasets generated for this study are included in the article/Supporting Information.
