# Supplementary material for: USP33 Regulates DNA Damage Response and Carcinogenesis Through Deubiquitylating and Stabilising p53
Source: Cell Prolif. 2024 Dec 18;58(5):e13793. doi: 10.1111/cpr.13793 (PMC12099211; doi:10.1111/cpr.13793)
Supplement: Supplementary file 1 — Figure S1. Knocking down of USP33 inhibits apoptosis in BEL7402. Figure S2. Knocking down of USP33 decreases the stability of mutant p53 R249S. Figure S3. Generation of hepatocyte‐specific USP33 knockout mouse (USP33‐HKO) strains. (A) Schematic diagram of generating USP33 knockout mice. (B) Breeding strategies for producing USP33 HKO mice. (C) Genotyping of wild‐type, heterozygous KO and HKO mice using PCR. (D) Western blot was performed to analyse USP33 knockout mice in liver tissues. [file CPR-58-e13793-s001.docx]

**Supplementary information**


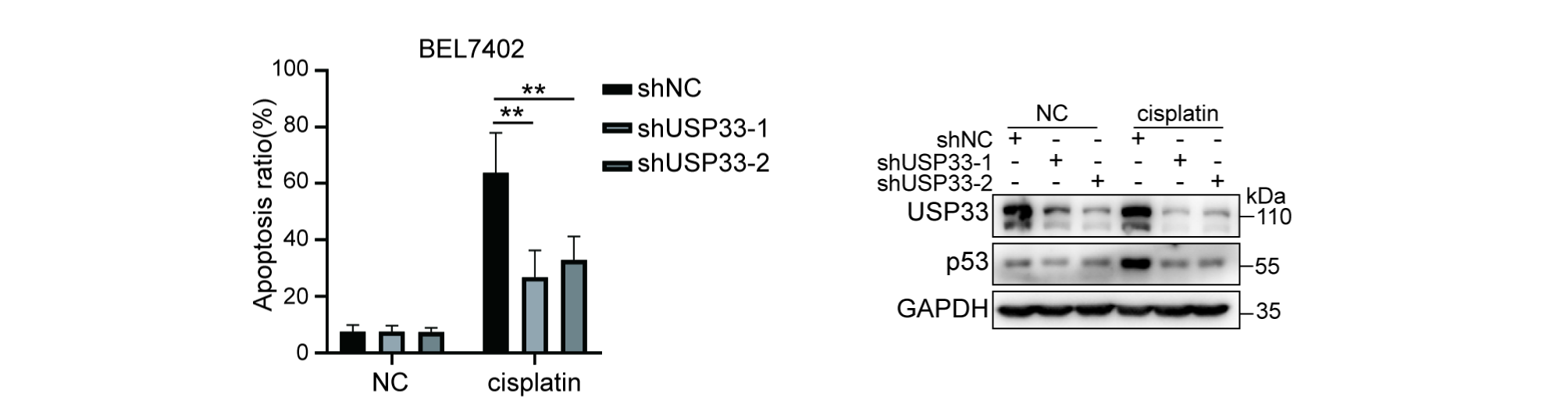


**Fig. S1 Knocking down of USP33 inhibits apoptosis in BEL7402.**


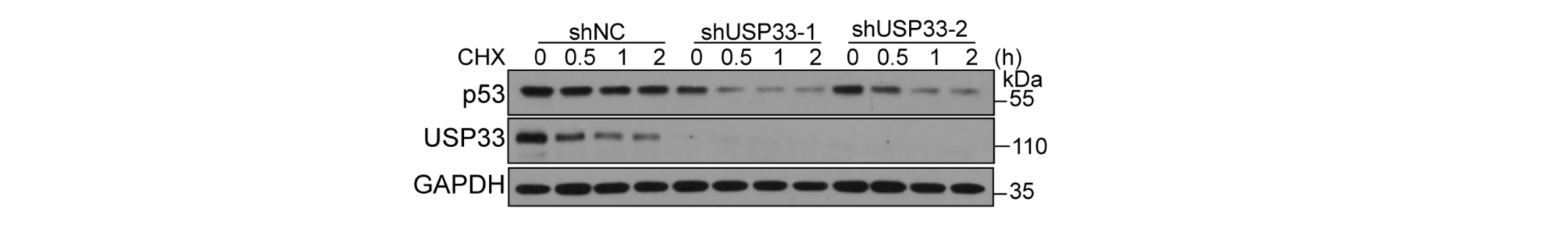


**Fig. S2 Knocking down of USP33 decreases the stability of mutant p53 R249S.**


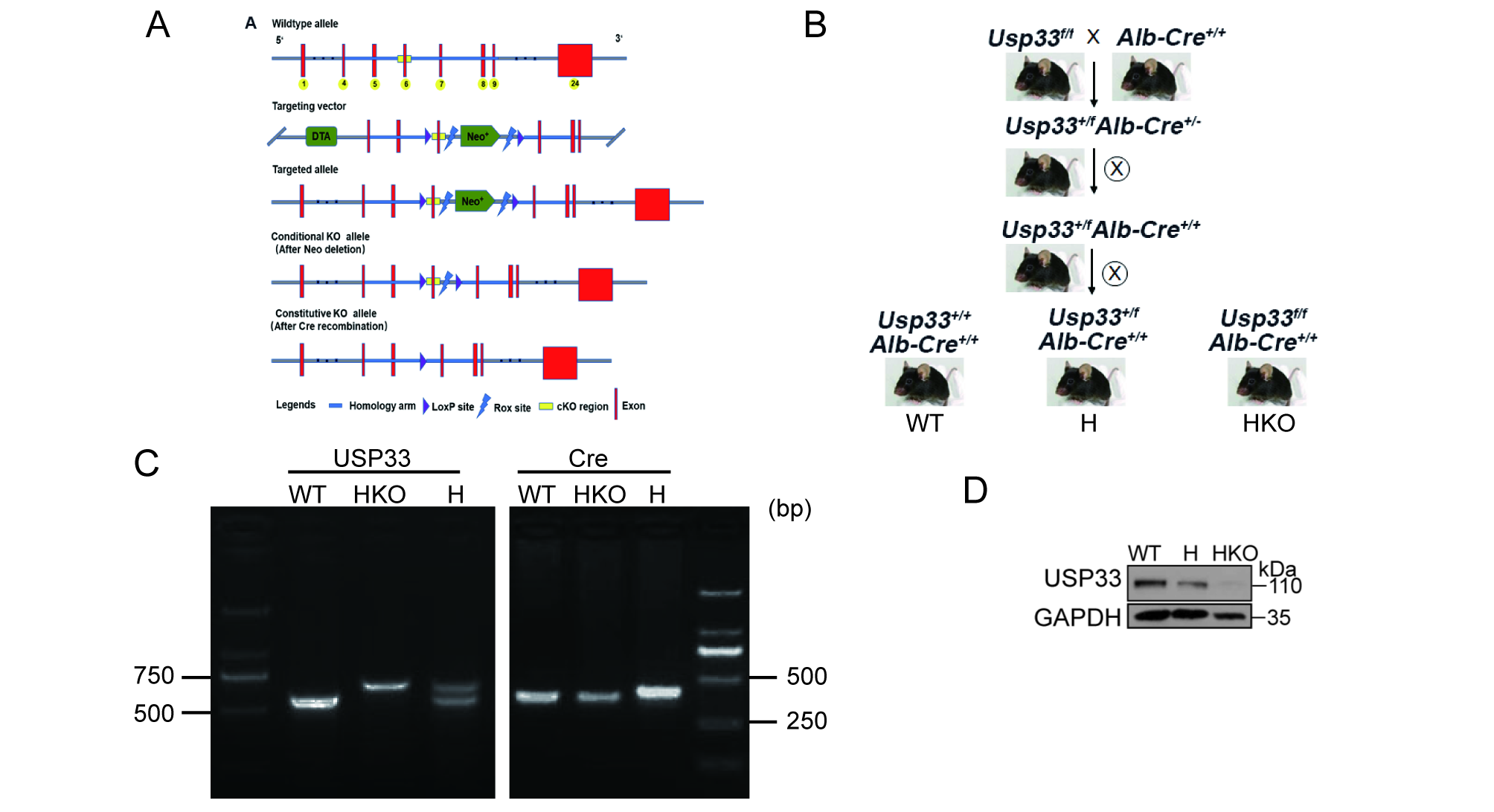


**Fig. S3 Generation of****hepatocyte-specific USP33 knockout mouse (USP33-HKO) strains.** **A** Schematic diagram of generating USP33 knockout mice. **B** Breeding strategies for producing USP33 HKO mice. **C** Genotyping of wild-type, heterozygous KO and HKO mice using PCR. **D** Western blot was performed to analyze USP33 knockout mice in liver tissues.
